# Differential Diagnoses of Cerebral Hemiatrophy in Childhood: A Review of Literature with Illustrative Report of Two Cases

**DOI:** 10.5539/gjhs.v5n3p195

**Published:** 2013-03-20

**Authors:** Uduma Felix U., Emejulu Jude-Kennedy C., Motah Mathieu, Okere Philip C. N., Ongolo, Pierre C., Muna W

**Affiliations:** 1 Department of Radiology, Faculty of Clinical Sciences, University of Uyo, Nigeria; 2 Neuro-Surgery Unit, Nnamdi Azikiwe University Teaching Hospital, Awka, Nigeria; 3 Neuro-Surgical Unit, Department of Surgery, University of Douala, Cameroon; 4 Department of Radiology, University of Nigeria Teaching Hospital, Enugu, Nigeria; 5 Department of Radiology, University of Yaounde, Cameroon; 6 Department of Medicine, University of Yaounde, Cameroon; 7 Polyclinic Bonanjo, Douala, Cameroon

**Keywords:** cerebral hemiatrophy, MRI, childhood, calvarium

## Abstract

Childhood cerebral hemiatrophy is an uncommon clinical entity. Its aetiologies are diverse but can generally be grouped into congenital and acquired. The congenital type is intrauterine in origin while the acquired type occurs early in life, usually before two year of life.

When childhood cerebral hemiatrophy occurs, it evokes a spectrum of compensatory calvarial sequlae. These include ipsilateral calvarial thickening, diploe widening, hyper-pneumatization of paranasal sinues/mastoids, elevation of petrous bone and small middle cranial fossa. MRI is very effective in high lightening brain atrophy, associated parenchymal changes and even the above enumerated skull changes.

Our two case reports of left hemi-cerebral atrophy in male Cameroonian children seen in our MRI practice aptly demonstrated some of the aforementioned radiological features of childhood cerebral hemiatrophy noted in literature review.

## 1. Introduction

In broad terms, atrophy connotes irreversible loss of tissue (Porettis et al., 2008). In similar context, cerebral hemiatrophy or unilateral brain atrophy is the end-stage of various pathologies culminating in atrophy or hypoplasia of a single cerebral hemisphere (Jacoby et al., 1997). Cognitive derangement, behavioural change, hemiplegia, seizures and emotional deficits are possible functional implications (Siren et al., 2005).

Cerebral hemiatrophy (CHA) is infrequently encountered in paediatric clinical practice (Goyal et al., 2009; Sharma et al., 2006). However it exist and could be primary or secondary as defined by Alpers and Dear in 1939 (Sharma et al., 2006). The primary (congenital) CHA could be interwoven or aptly called cerebral hemi-hypoplasia or unilateral cerebral hypoplasia as it could actually be de-novo lack of cerebral development (Goyal et al., 2009; Sharma et al., 2006). Here, the insult occurs in-utero, with consequent shift of midline structures towards the side of the disease and absence of sulcal prominence (McMonagle et al., 2006). These features differentiate it from secondary CHA which could originate from cerebrovascular lesion, inflammatory process, or cranial trauma (Sharma et al., 2006).

Possible aetiologies of cerebral hemiatrophy are the followings:

(A) Congenital
  ○ Idiopathic
  ○ Intrauterine vascular injury
(B) Acquired
  ○ Birth trauma
  ○ Perinatal intracranial haemorrhage
  ○ Rasmussen encephalitis
  ○ Postictal cerebral hemiatrophy, Prolonged febirile Seizures
  ○ Infection like Herpes Encephalitis, TORCH syndrome, HIV
  ○ Vascular/Haematological abnormalities like Dyke Davidson Mason syndrome, Sturge-Weber syndrome, Crossed Cerebral Cerebellar Diaschiasis, Diaschiasis Commisuralis, Hemiplegia-Hemiatrophy Hemiconvulsion syndrome
  ○ Ischaemia
  ○ Neoplasia like basal ganglial germinoma
  ○ Radiation
  ○ Phakomatosis (Neurofibromatosis)
  ○ Miscellaneous-Linear Nevus syndrome, Fishman syndrome, Silver-Russell syndrome, Infantile hemiplegia syndrome, Congenital malformation, Intrauterine coactation of aorta, Perinatal anoxia/hypoxia; Mitochondrial encephalopathy, lactic acidosis, and stroke-like episodes (MELAS) (Goyal et al., 2009; Sharma et al., 2006; Radswiki & Gaillard, 2011; Chakravar, 2002; Singh et al., 2002; Salisu & Awosanya, 2010).

Neuroimagings like computed tomography (CT) and magnetic resonance imaging (MRI) play key diagnostic role in paediatric neurology. MRI is the preferred modality in assessment of the aetiology and lesion extent of cerebral parenchyma in atrophy, seizures, hemiparesis/plegia, and craniofacial asymmetry (Facial hemiatrophy, 2012; Atalar et al., 2007). Pathologic accompaniment in brainstem and cerebellar hemisphere are further elaborated by MRI (Porettis et al., 2008; Facial hemiatrophy, 2012).

## 2. Aims

To emphasis the imaging features of some of the differential diagnoses of childhood cerebral hemiatrophy with illustrative corroborations from two case reports.

## 3. Case Reports

### 3.1 Case 1

AA, a 15year old Cameroonian boy whose symptomatology dates back to 2 year of life. This was due to coma precipitated by febirile attack leading to poor learning ability and speech. He was later observed by his parents to have abnormal rapid growth with precocious hirsutism. Overseas referral was then made due to local non-availability of neuro-imaging tools. A diagnosis of Gigantism with left unilateral cerebral atrophy was made. He was placed on monthly Bromocriptine therapy to control growth.

**Figure 1:**
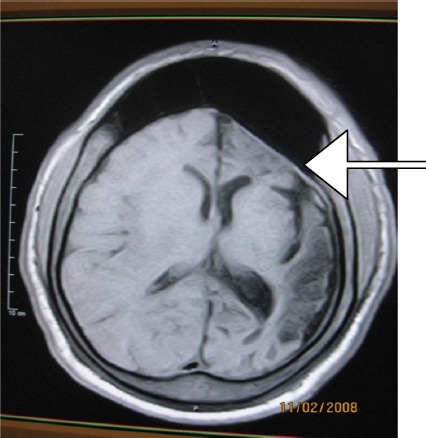
T1W axial MR image showing left hemicerebral atrophy with hypointense parietal lesion, enlargements of frontal sinus and left atrium

**Figure 2:**
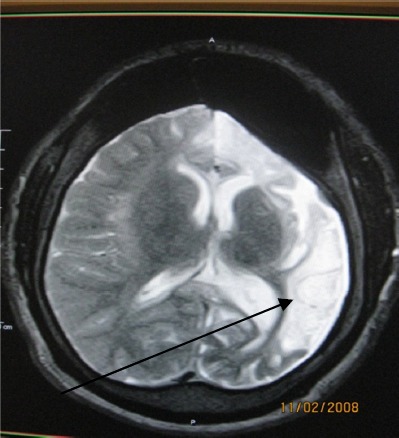
T2W axial MR image delineating a hypointense thin wall of a hyperintense left parietal lesion with surrounding hyperintense infarction

**Figure 3:**
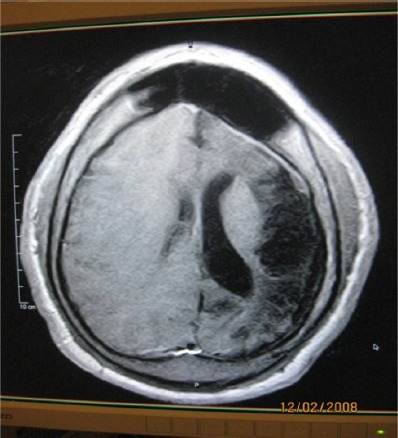
Enhanced axial T1W image of Figure 2 showing rim enhancement of above lesion

**Figure 4:**
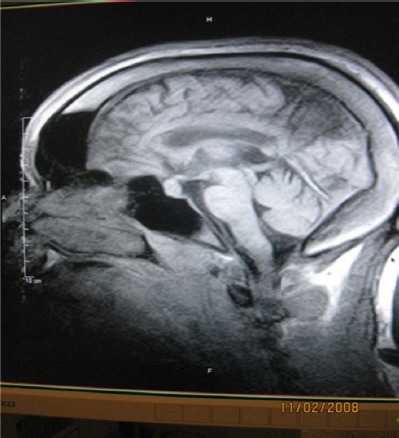
Sagital enhanced T1W image showing pituitary microadenoma and enlargement of diploe, frontal and sphenoidal sinuses

**Figure 5:**
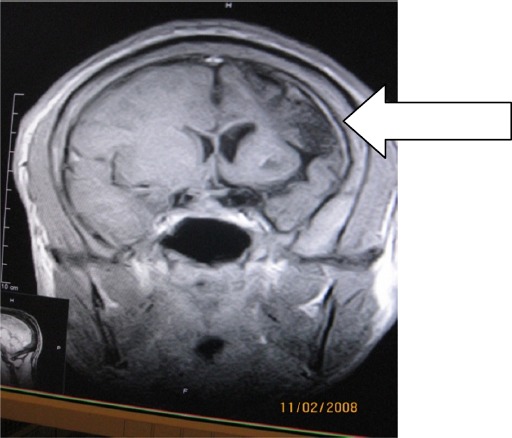
Coronal enhanced T1W image showing left hemicerebral atrophy with ipsilateral frontal horn enlargement and left parietal lobe lesion

**Figure 6:**
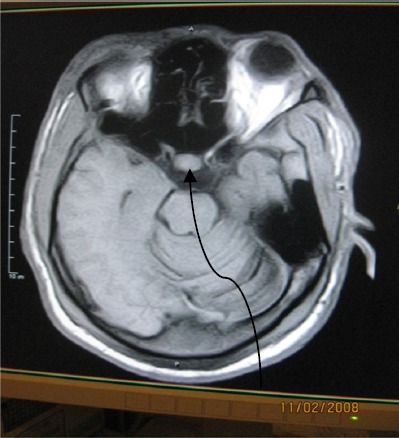
Axial MRI T1W image showing pituitary microadenoma, atrophic left temporal lobe with small tempoal fossa and enlarged mastoid air cell

**Figure 7:**
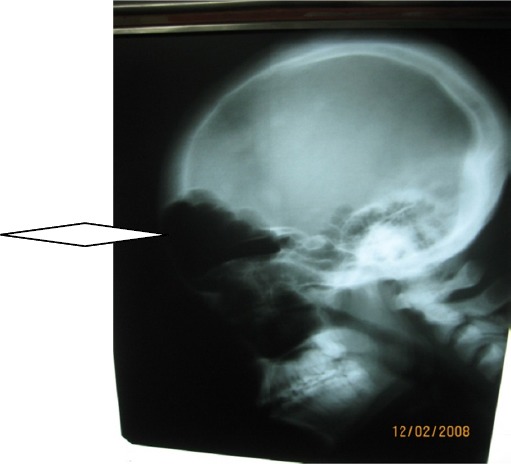
Lateral skull radiograph showing enlarged frontal and maxillary sinuses

MRI done in our hospital following recurrent seizures for six months showed left cerebral atrophy with predominance in frontal and temporal lobes (Figures 1 & 2). This was associated with left parietal lobe T1W hypointense and T2W hyperintense lesion with rim enhancement implicating encephalomalacia or cerebral abscess. Also Pituitary Microadenoma was found on MRI with identification of intra-sellar enhancing solid well defined mass less than 1cm in diameter. This was best depicted on axial, sagital and coronal sections (Figures 3, 4, 5 and 6). Secondary changes of focal ventriculomegaly of left frontal horn as well as homolateral diploe/inner table hypertrophy, small middle cranial fossa and hyperpneumatization of frontal/sphenoid sinuses were seen. This was confirmed by skull radiographs (Figure 7).

### 3.2 Case 2

CA, is a 12year old Cameroonian boy whose parents were divorced on account of his ailment. Birth was uneventful but he was said to have developed right index finger weakness and slurred speech at 0-3years. These improved with age (5) due to exposure to peer group and television. Recently, he became hyperactive and developed unexpected epileptic attacks in school. There was no history of delayed mile stones, detection of neurocutaneous markers nor facial asymmetry. At age 5, brain CT showed left brain atrophy. Brain MRI four months later confirmed left unilateral cerebral atrophy.

Recent brain MRI in our centre showed severe atrophy of left cerebral hemisphere with the left temporal lobe and inferior frontal lobes being worst affected. Heavy T2W TR/TE 4000/120 axial images also showed supra-ventricular left parietal lobe 32 × 17mm wedge shape hyperintense lesion. Similar 43.9 × 15.6 T2W high signal lesions was seen at the mid-ventricular left temporo-parietal lobe. These lesions have peripheral grey mater base and extend into sub-cortical fibres. They show subtle gyriform GD-DTPA enhancement but without intravascular enhancement sign. FLAIR sequences showed these lesions to be isointense to CSF. Observed consequences of unilateral contraction of left cerebral hemisphere especially the temporal and inferior lobes from the inner table were (1) decrease in the relative size of left basal ganglia (2) little interposed coronal radiata between ventricles and deep cortical sulci (3) mild ex-vacuo enlargement of frontal horn and left transverse sinus (4) elevation of the petrous ridge and reduced size of the middle cranial fossa.

A diagnosis of Left unilateral cerebral atrophy (Intrauterine Herpes Encephalitis) with supervening Encphalomalacia was made. Conservative management led to significant remission of symptoms.

## 4. Discussion

Cerebral hemiatrophy (CHA) is an entity with diverse aetiologies and pronounced asymmetry of cerebral hemispheres (Vossakamper & Schachenmayer, 1990). It may be a progressive spectrum or emanating from a single event (Porettis et al., 2008).

Half and three-fourths of adult brain sizes are attained during the first and third year of life respectively (Sharma et al., 2006). With brain growth, the brain presses outward on the calvarial tables underscoring the gradual enlargement and general shape of the adult head (Sharma et al., 2006). Identification of compensatory calvarial changes presuppose that cerebral abnormalities are the consequences of an atrophic or hypoplastic process from brain insults that started in early life (usually before 2 years). These skull changes correlate with the amount of underlying brain atrophy. These changes include thickening of the calvarium with loss of convulational markings of the inner table and flattening of calvarium (recognisable on skull radiograph-Figure 7). Overpneumatization of paranasl sinuses and mastoid air cells lead to elevation of the petrous ridge (Jacoby et al., 1997) (See Figures 1, 3, 4, 5).

Incidentally, our two patients have left unilateral cerebral atrophy, just as Atalar et al detected left cerebral predominance in 14 out of 19 cases studied (Atalar et al., 2007). Hyperpneumatization as seen in our patient was also seen in 26.11% of their patients. The reason for the recalcitrant seizures and poor intellect seen in our first patient is not far-fetched considering the greater volume of cerebral atrophy and supervening encephalomalacia/abscess. Children with cystic encephalomalacia plus atrophy have poorer prognosis than children with atrophy or gliosis alone (Bava et al., 2007). Predictors of intellectual functions are morphometric variables like volumes of cortical grey mater, integrity of white mater and lesion size (Bava et al., 2007).

ur second patient showed less constellation of compensatory calvarial changes impling late onset of CHA. The propensity of Type 11 Herpes simplex for temporal lobe, frontal lobe and limbic system raised the suspicion of herpes simplex viral infection in our second patient (Dahnert, W, 2007). However, absence of any unilateral calvarial thickening and sinus pneumatizations in this second patient rules out Dyke Davidson Mason syndrome. Likewise, the lesions being hyperintense on T2W and not isolated to deep white mater rules out Demyelinating diseases like Peliazeus Merzbacher disease. Fine peripheral arborizations of the white mater tracts and high signal of corpus callosum on T1W supports normal myelinations and brain maturation. No peri-ventricular nor basal ganglia T1W and T2W low signal foci to suggest granulomatous calcifications or Neurofibromatosis

### 4.1 A Cursory Look at Some of the Aetiologies of Cerebral Hemiatrophy

(1) Dyke Davidoff Mason Syndrome

In 1933, Dyke, Davidoff and Masson described skull radiographic and pneumatoencephalographic changes in a series of nine patients characterized clinically by hemiparesis/hemiplegia, seizures, facial-asymmetry, and mental retardation (Sharma et al., 2006; Singh et al., 2002). The aforementioned are the hallmark clinical presentations of Dyke-Davidoff-Masson syndrome (DDMS). DDMS or hemispheric infarction is a childhood unicerebral atrophy or hypoplasia with compensatory ipsilateral calvarial hypertrophy (Goyal et al., 2009; Sharma et al., 2006; Chridtopher-Rodgman et al., 2011). This infrequent condition is secondary to brain insult in-utero or early childhood period. Insult could be vascular, congenital or acquired ischaemic disease, trauma or inflammation (Goyal et al., 2009; Sharma et al., 2006). In-utero gestational vascular occlusion involving middle cerebral vascular territory or coactation of mid-aortic arch may decrease carotid arterial flow (Goyal et al., 2009; Sharma et al., 2006; Singh et al., 2002).

In DDMS, calvarial changes occur only when brain damage is sustained before three years of age but such changes may become visible as early as nine-months after brain damage (Sharma et al., 2006). Brain growth failure compels inward redirection of nearby calvarial growth accounting for the compensatory calvarial changes like enlargement of the frontal sinus, increased width of the diploic space with elevations of the ipsilateral greater wing of sphenoid, petrous ridge and planum sphenoidale (Sharma et al., 2006; Facial hemiatrophy, 2012; Chridtopher-Rodgman et al., 2011). CT is preferred to conventional radiography in DDMS as skull plain radiographs may be so subtle and overlooked (Sharma et al., 2006; Radswiki & Gaillard, 2011).

MRI features in DDMS include variable extent of unilateral loss of cerebral volume (with or without enlargement of the cortical sulci and perimesencephalic cistern), hypoplasia/atrophy of the cerebral peduncle, abnormal myelination and contralateral cerebellar atrophy (Sharma et al., 2006; Chakravar, 2002; Facial hemiatrophy, 2012). While the later is due to postictal lobar sclerosis, hypoplasia of the ipsilateral mesencephalon is due to distal axonal degeneration (Salisu & Awosanya, 2010).

Treatment is symptomatic but intractable disabling seizures and hemiplegia may be indications for hemispherectomy. This eliminates or remarkably reduces seizures in 85% of patients (McMonagle et al., 2006; Salisu & Awosanya, 2010). Better prognosis obtains in DDMS with hemiparesis coming after age two or absence of prolonged or recurrent seizures (McMonagle et al., 2006).

(2) Herpes Simplex Encephalitis

The causative organism in neonates and infants is Herpes simplex virus (HSV) type 11 and transmission is either transplacental or at birth (Vossakamper & Schachenmayer, 1990; Moritani, 2011). Herpes simplex encephalitis (HSE) is a non-epidermic, necrotizing meningo-encephalitis with haemorrhagic necrotic tendency, considerable mass effect and rapid dissemination in brain (Vossakamper & Schachenmayer, 1990; Moritani, 2011).

HSV intrauterine fetal brain infections can either cause cerebral hemiatrophy or microcephaly (Moritani, 2011). Hemiatrophy is due to initial unilateral involvement with propensity for limbic system (olfactory tract, temporal lobes, cingulate gyrus, insular cortex) (Vossakamper & Schachenmayer, 1990) (Like our second patient). Descending order of involvement is inferior-medial temporal lobe >frontal>parietal lobes (Dahnert, 2007). MRI is cardinal in neonatal HSE evaluations as both cortex and white matter are extensively involved (Marisa & Glenn, 2007). CT becomes more valuable in calcifications mainly periventricular and grey/white mater junction (Moritani, 2011). CT features of HSE are poorly defined hypo-attenuated areas in temporal lobes with sharply defined concave/straight border sparing putamen (Vossakamper & Schachenmayer, 1990). Lesions are earlier identified in MRI and seen to be more extensive than in CT due to MRI greater sensitivity to white mater changes. HSE appear as high signal on T2W images and mild to moderate hypointensity on T1W (Moritani, 2011). Small foci of haemorrhage are common and may occur as increased signal on T2W images and usually implies extensive necrosis (Vossakamper & Schachenmayer, 1990; Moritani, 2011). Enhancement pattern are mildly patchy, peripheral, gyral or cisternal (Vossakamper & Schachenmayer, 1990).

Identification of virus within CSF (using polymerase chain reaction) or viral culture from brain biopsy is diagnostic (Vossakamper & Schachenmayer, 1990). Treatment is Adenine arabinoside.

(3) Sturge Weber Syndrome

Sturge-Weber syndrome (SWS) or encephalofacial/encephalotrigeminal angiomatosis, is a rare, congenital neurocutaneous syndrome accompanied by unilateral facial cutaneous vascular malformation (nevus flammeus or port-wine stain {PWS}) and ipsilateral leptomeningeal angiomatosis (Jacoby et al., 1997; Marisa & Glenn, 2007; Kelly et al., 2005; Omran et al., 2005). Paucity of superficial cerebral cortical veins with malformed fibrotic veins usually ipsilateral to the skin lesion is the underlying anomaly. Prevalence is approximately one per 50 000 live births with equal sex ratio and no racial bias (Kelly et al., 2005). The facial PWS is usually purple to pink in appearance and involves one side of upper face, including the eye corresponding to ophthalmic divisions of the trigeminal nerve. Maxillary and mandibular facial trigeminal skin area PWS could also be seen (Omran et al., 2005). Other manifestations include progressive seizures, ipsilateral glaucoma, ipsilateral cranial thickening, contralateral hemiparesis, hemiatrophy, hemianopia and mental retardation (Jacoby et al., 1997; Sharma et al., 2006; Omran et al., 2005). Clinical neurologic impairment is not influenced by PWS rather by the greater anatomic hemispheric manifestations (Omran et al., 2005; Lin et al., 2008). Cerebral atrophy in SWS are typically unilateral and confined to the parieto-occipital area, but occasionally may extend to the entire hemisphere or bilaterally (Marisa & Glenn, 2007; Kelly et al., 2005; Omran et al., 2005). Abnormality of the cerebral parenchyma can be detected from birth in some patients and has a progressive character of atrophy and parenchymatous hyper density of the affected hemisphere, as well as a decrease in arterial size, especially during the first decade of life (Lin et al., 2008). MRI usually shows evidence of ipsilateral tissue loss with hallmarks of tubular regions of flow void and gyriform hypointensities on T1- and T2-weighted MRI images due to venous collateralization and cortical calcification (Marisa & Glenn, 2007). These hallmarks show on CT as “tram-line” or gyriform calcifications usually involving the occipital and parietal lobes underlying the leptomeningeal angiomatosis (Marisa & Glenn, 2007). The cortical calcification may be as a result of chronic hypoxic injury (Kelly et al., 2005). Histology has revealed these intracranial lesions are leptomeningeal angiomatosis, Leptomeningeal thickening, gyriform calcifications, neuronal loss, astrogliosis in underlying brain tissue, choroidal angioma, and choroid plexus angioma. (Zhou et al., 2010). Calcification is seen histologically in almost every case, 90% in CT and 50-60% in skull radiographs (Jacoby et al., 1997; Kelly et al, 2005). Bilateral calcifications occurred in 15% of cases with skull thickening seen on the side of more extensive calcifications (Jacoby et al., 1997). However, calcifications may be absent or minimal in neonates and infants (Zhou et al., 2010).

MRI with contrast is the preferred imaging modality for the identification of structural brain abnormalities in SWS (Zhou et al., 2010). It shows enhancement of the leptomeninges overlying the involved cortex, reflecting leptomeningeal angiomatosis, or blood-brain-barrier damage related to chronic cortical ischemia. Homogeneous enhancement of an enlarged ipsilateral choroid plexus may be seen due to a choroid plexus angioma. Because of insufficient superficial cortical venous drainage, collateralization develops in the deep venous system, leading to enhancement of medullary and subependymal veins ipsilateral to the cortical lesion (Zhou et al., 2010). MR perfusion weighted imaging (PWI) indicates cerebral hypoperfusion predominantly due to impaired venous drainage, with only the most severely affected regions in some patients also showing arterial perfusion deficiency (Lin et al., 2008). Functional neuroimaging studies with positron emission tomography (PET) and single photon emission computed tomography (SPECT) may demonstrate cortical hypometabolism and hypoperfusion in corresponding area.

(4) Rasmussen Encephalitis

Rasmussen syndrome (RS) or encephalitis or chronic focal encephalitis was discovered in 1958 by Theodore Rasmussen (Salisu & Awosanya, 2010; Bien et al., 2005). It is a rare but severe immune-mediated, chronic inflammatory, progressive, devastating brain disorder (Omran et al., 2005; Lin et al., 2008; Bien et al., 2005; Zhang et al., 2007; Wikipedia, 2010; Tien et al., 1992). The onset is in childhood and is characterized by unilateral hemispheric atrophy, abrupt appearance of focal, persistent motor seizure activity (epilepsia partialis continua {EPC}), followed by hemiplegia and progressive cognitive deterioration (Zhang et al., 2007). Three features of the epilepsy in RE are polymorphism of seizures, frequent occurrence and medical intractability. (Bien et al., 2005). RS is usually uni-cerebral and generally occurs in children under the age of 15 with median age of 6years (Salisu & Awosanya, 2010; Bien et al., 2005; Wikipedia, 2010). Nevertheless, adolescent and adult patients with milder course exist (Ouesadam et al., 2007; Bien et al., 2005).

Aetiology of RS is unknown but autoimmunity associated with persistent viral infection has been implicated (Moritani, 2011; Wikipedia, 2010; Tien et al., 1992). Preceding inflammatory episodes are tonsillitis, upper respiratory tract infection, and otitis media (Salisu & Awosanya, 2010). Unihemispherical atrophy results from brain cells inflammations and epilepsy (Sharma et al., 2006). Since in RE the brain involvement is mainly unilateral, some factor additional to autoimmunity must contribute to the pathogenesis in order to determine unilaterality (Bien et al., 2005; Wikipedia, 2010).

RS is a diagnosis of exclusion and typically insidious in onset (Salisu & Awosanya, 2010; Tien et al., 1992). CT, xenon CT, PET and MRI will all show unihemispheric lesion. CT and MR revealed nonspecific atrophy, xenon CT showed decreased cerebral blood flow, and PET revealed a hypometabolic state (Zhang et al., 2007; Tien et al., 1992). MRI demonstrates the progression of RE and may suggest the diagnosis in the early stages, prior to appearance of neurological deficits. Apart from atrophy of the head of the caudate nucleus, MRI may also show associated secondary changes such as atrophy of the contralateral cerebellar hemisphere, ipsilateral hippocampus, and brainstem (Salisu & Awosanya, 2010). In the first 4 months after disease onset, the majority of patients exhibit unilateral enlargement of the inner and outer CSF compartments, most accentuated in the insular and peri-insular regions, with increased cortical or subcortical (or both) T2W and FLAIR signals (Bien et al., 2005). Large increase in MRI FLAIR signal suggests active inflammation or strong astrogliosis of the affected brain. One characteristic and puzzling property of Rasmussen encephalitis is respect of the midline of the cerebral hemispheres (Ouesadam et al., 2007). Tissue loss occurs during the first 12 months after onset of the acute disease stage, however, it may, in some cases, go on for several years (Bien et al., 2005). There are four recognized stages of RE based on T2 weighted MRI criteria (Kelly et al., 2005). These are swelling with hyperintense signal (stage 1); normal volume with hyperintense signal (stage 2); atrophy with hyperintense signal (stage 3); and progressive atrophy and normal signal (stage 4) (Salisu & Awosanya, 2010). Gadolinium enhancement is very rare (Marisa & Glein, 2007).

PET guides brain biopsy in early cases, inconclusive or normal MRI findings. PET with fluorodeoxyglucose (FDG-PET) detect changes in early stages (disease duration up to 1 year) with lesions confined to frontotemporal areas, affecting posterior cortical regions in later stages (Bien et al., 2005). Ictal SPECT is useful for the localization of the epileptogenic focus in the affected cerebral hemisphere (Salisu & Awosanya, 2010).

Atypical RS are dual pathologies with low grade tumour, cortical dysplasia, tuberous sclerosis, vascular abnormalities or old ischaemic lesions. Bilateral cerebral affection is due to unaffected hemisphere undergoing milder form of atrophy from Wallerian degeneration of commissural fibres, chronic epilepsy or treatment. True bilateral simultaneous RE is very rare, fatal and has poor prognosis (Salisu & Awosanya, 2010; Bien et al., 2005).

Antiepileptic drugs are not effective for RS. Immunomodulatory treatment with either high-dose steroids or intravenous immunoglobulin is treatment option and can achieve more than 50% reduction in seizure frequency (Salisu & Awosanya, 2010; Moritan, 2011; Zhang et al., 2007). Functional hemispherectomy, remains the only curative treatment with focal cortical resection as alternative (Salisu & Awosanya, 2010).

(5) Crossed Cerebellar Diaschisis

Diaschisis is reduced function of a brain section because of interruption at a remote site of an afferent pathway which normally supply background excitation to the neurons in that section. This concept was initially invented by von Monakow who described diaschisis corticospinalis (progression of functional depression of the spinal cord after an injury to the motor cortex); diaschisis commisuralis (functional depression of the contralateral cerebral cortex following injury to a hemispherical cortex); and diaschisis associativa (depression of function in intact cortical areas adjacent to the site of a cortical injury). Others are subcortical-cortical diaschisis or transcallosal diaschisis. The concept of crossed cerebral cerebellar diaschisis (CCCD) after injury (usually vascular) to the motor cortex of one cerebral hemisphere was popularised by Baron et al in 1980 using PET images to exhibit contralateral cerebellar hypometabolism following supratentorial infarcts (Singh et al., 2002).

Cerebellar hemisphere is connected to the contralateral cerebral cortex via feed-back circuits, required for smooth execution of motor function. CCCD is usually associated with long standing, extensive and unilateral cerebral lesions with onset during infancy or early childhood like DDMS, Parry Romberg syndrome cerebral infarction, demyelination, post-hemorrhagic conditions, infantile hemiplegia syndrome, supertentorial ictus epilepsy, Rasmussen encephalitis, extreme prematurity and neoplasm like basal ganglial germinoma (Jacoby et al., 1997; Chakravar, 2002; Marisa & Glenn, 2007; Ouesadam et al., 2007). Diaschisis may manifest as ipsilateral or contralateral depending upon the age of cerebral insult. CCCD is uncommon in children (Marisa & Glenn, 2007). Early insults would likely be ipsilateral and later ones likely a CCCD (Singh et al., 2002).

Damage to the corticopontocerebellar pathway or cerebellorubrothalamic tract with transneuronal hypometabolism is the most acknowledged pathogenic mechanism in the development of CCCD (Singh et al., 2002; Marisa & Glenn, 2007). The immature cerebellum is presumably dependent on the trans-synaptic excitatory pathways for normal growth and development (Marisa & Glenn, 2007). CCCD is a based on Wallerian degeneration (degeneration of the myelin sheath and axon distal to the most proximal site of axonal interruption secondary to axonal disease) (Singh et al., 2002). MR reveals the structural changes induced in the cerebellum resulting from such changes in vascularity and metabolism (Facial hemiatrophy, 2012). Some correlation exists between the degree of morphologic damage to the cerebrum and the volume loss in the opposite cerebellum. Wallerian degeneration may be seen on MRI as a hyperintense signal on the T2W images. At 4 to 5 weeks, a well-defined band of hypointensity (resulting from transitory increased lipid-protein ratio) may appear on the T2W images in the topographic distribution of the connecting fibres in ipsilateral cerebral peduncle/pons (suggesting degeneration of ipsilateral cortico-bulbar and cortico-spinal tracts) and contralateral cerebellum). Signal becomes permanently hyperintense after 10-14 weeks. Signal alterations are usually profound at about 3-6 months. Shrinkage of ipsilateral brainstem and cerebellum may appear by 8 months (Singh et al., 2002).

(6) Peri-Natal Asphyxia

Vosskamper et al reported a case of primary cerebral hemiatrophy with severe white mater lesions following perinatal asphyxia as neurological symptoms were obvious immediately after birth (Vossakamper & Schachenmayer, 1990).

(7) Parry Romberg Syndrome

Parry-Romberg syndrome (PRS) or progressive hemifacial atrophy (PFH), was first described by Parry in 1825 then Romberg in 1846. PRS is a poorly understood rare disorder of possible neurovascular origin characterized by hyperpigmentation of skin, unilateral wasting of the cranio-facial skin and subcutaneous tissue with variable involvement of underlying musculoskeletal and neural tissues (Facial hemiatrophy, 2012; Fry et al., 1992). Hereditary factors have been implicated in the aetiology of PFH but queried by some authors who reported the occurrence of PFH in one of monozygotic twin pair possibly ruling out genetics (Facial hemiatrophy, 2012).

Clinical manifestations are epilepsy, migraine, hemiplegia, ptosis, enophthalmos, unilateral hypoplasia of the face and flattening of the curvature of the calvarium (Fry et al., 1992, Jacoby et al., 1997). Mental retardation may or may not be present and there may be shortening of the extremeties on the affected side (Jacoby et al., 1997).

MRI is more sensitive than CT in the diagnostic evaluation of patients with PFH (Verhelst et al., 2008). Findings on brain MRI are usually ipsilateral to the facial hemiatrophy and include leptomeningeal enhancement, diffuse areas of high signal intensity in the white matter, unilateral focal infarctions in the corpus callosum, brain stem involvement and multiple intracranial aneurysms (Fry et al., 1992). Jacoby et al. (1997) found 4 PRS patients with hemiplegia/convulsive disorders and their CT findings were unilateral cerebral loss of volume with ipsilateral displacement of midline structures. A case also showed ipsilateral falx displacement indicating a process that commenced before or during the time of greatest growth of the calvarium, thus excluding a contralateral lesion acquired later in life.

Treatment is symptomatic and consists of plastic surgery after the disease activity has ended (Jacoby et al., 1997).

PFH usually occurs in the first two decades of life and the clinical presentation resembles linear scleroderma (Fry et al., 1992). Other differentials are Sturge-Weber syndrome and linear sebaceous nevus syndrome (Jacoby et al., 1997).

(8) Linear Sebaccious Nervus Syndrome

Linear sebaccous nevus syndrome is a neurocutaneous condition similar to tuberous sclerosis with mental retardation, convulsions and characteristic linear nevus of the face. Jacoby et al. (1997) reported a case with CT showing skull asymmetry, unilateral left ventriculomegaly/hemiatrophy and the other ventricles were normal. Skull radiograph showed elevation of the left petrous ridge, extensive erosion of the sella turcica and body of the sphenoid bone. The recognition of compensatory calvarial changes presupposes that the atrophic or hypoplastic process began in early life (Jacoby et al., 1997).

(9) Linear Scleroderma

Scleroderma (Progressive systemic sclerosis) is a multi-system connective tissue disorder of unknown aetiology. It is characterized by widespread disorder of the micro-vasculature and over-production of collagen eliciting exuberant interstitial fibrosis, sclerosis and atrophy of many organ system (Vossakamper & Schachenmayer, 1990). Linear scleroderma called en coup de sabre, is characterized by linear atrophy of the forehead likened to a saber strike (Fry et al., 1992). Linear scleroderma of childhood and hemifacial atrophy have significant clinical overlap and they seem to be manifestations of the same or related pathogenicity (Moritani, 2011).

Linear scleroderma should be included in the differential diagnosis in patients with unilateral hippocampal atrophy even when the typical skin lesions are not present (Moritani, 2011).

1.25-dihydroxyvitamin D3 (calcitriol) may be an effective agent for treating localized scleroderma in children (Facial hemiatrophy, 2012).

(10) Status Epilepticus

In status epilepticus, neuronal injury probably results primarily from an excitotoxic mechanism mediated by intrinsic neuronal seizure activity. In status epilepticus, neuronal seizure activity increases release of glutamate from the pre-synaptic terminal of neuronal axons. Glutamate crosses the synaptic cleft to bind to receptors, leading to prolonged depolarization and resultant apoptosis or necrotic cell death. Seizures further exacerbate ischemic and metabolic compromise of brain parenchyma resulting in further injury and unilateral brain atrophy (Marisa & Glenn, 2007).

Encephalopathy with status epilepticus often involves the hippocampus, other parts of the limbic system, thalamus, and cerebellum (Marisa & Glenn, 2007).

(11) Post-Ictal Cerebral Hemiatrophy

Vossakamper and Schachenmayer (1990) reported a case of postictal cerebral hemiatrophy with a wide spread loss of cortical neurons of the entire single hemisphere. This is notably a secondary cerebral hemiatrophy as the disease process began at age of two years after a widely normal early development.

(12) Neurofibromatosis

Unilateral cerebral atrophy in Neurofibromatosis (NF) is accompanied by compensatory hypertrophy of contralateral cerebrum. A potential pitfall is presuming that the hypoplastic cerebrum is the anomaly in NF. Hemimegalencephaly, gliomatosis cerebri, widespread cortical dysplasia are other NF features (Dahnert, 2007).

(13) Head Trauma

Vascular disruption or wallerian degeneration may be the aetiogenesis of unilateral cerebral atrophy in infantile head trauma (Jacoby et al., 1997).

(14) Hemiconvulsion-Hemiplegia-Epilepsy Syndrome (HHE)

Parenchymal damage results from sustained ictal activity in HHE. Few cases of HHE patients have been reported in literature. An example is a two-year and nine-month-old boy who had a prolonged hemiconvulsion during fever followed by right hemiparesis. After seven days, T2W and diffusion weighting imaging abnormalities were restricted to the white mater of the left hemisphere. Follow-up MRI in subsequent month showed severe gliosis and unilateral brain atrophy (Toldo et al., 2007).

(15) Brain Tumours

Ipsilateral brain atrophy is rare in brain neoplastic lesions (Wong et al., 1991). However, cerebral hemiatrophy has been reported in neoplasia like germ cell tumours located in pineal gland, suprasellar region, basal ganglia and thalamus (Wong et al., 1991; Sartori et al., 2007; Ozalame et al., 2006). Others are low grade astrocytoma, pinealoma, gliobastoma (Mutoh et al., 1988; Jayakuma et al., 1983). Brain tumors with secondary ipsilateral CHA are so rare that Maehara et al on literature review reported a total of ten cases including their own three new discoveries (Mutoh et al., 1988). Tamaki et al. (1990) reviewed forty-two cases (including their two new cases) of ipsilateral CHA due to basal ganglia germ cell tumours. Mutoh et al. (1988) investigated CT findings of 19 children under 16 years of age with primary brain tumors in the cerebral hemisphere with co-existing ipsilateral CHA. Ipsilateral CHA was observed in 21% of their cases shared equally by germinoma in the basal ganglia and low-grade astrocytoma in the frontal and occipital cortex (Mutoh et al., 1988).

Cerebral germinomas is the most common and least malignant intracranial germ cell tumors usually found in the midline (Sartori et al., 2007; Ozalame et al., 2006). They usually arise in the pineal or suprasellar region with characteristic clinical and radiological features but rare in the thalamus, basal ganglia, and internal capsule (Sartori et al., 2007). Basal ganglia germinoma called ectopic germinoma is rare and represent 5% to 10% of all intracranial germinomas (Ozalame et al., 2006). It is suggested that germinoma should be included in the differential diagnosis of a haemorrhagic mass in the thalamus, basal ganglia, or internal capsule which is associated with cerebral hemiatrophy and progressive hemiparesis especially in adolescents or young adults (Lee et al., 2008; Kim, 2002).

Hemiatrophy preceding or accompanying the imaging findings of a basal ganglia mass lesion is thought to be caused by a paraneoplastic process (Ozalame et al., 2006). Ipsilateral CHA is induced by Wallerian’s and retrograde degenerations as a consequence of the secondary damages of the thalamic ganglion cells, afferent and efferent nerve fibers from tumour invasiveness (Kim & Suzuki, 1975).

The major symptoms of basal ganglia germ cell tumours are hemiparesis, mental deterioration, precocious puberty, diabetes insipidus, personality change, oculomotor palsy, declining school work, speech disturbance, and hemianopsia (Kwak & Suzuki, 1975; Lin et al., 2006) The association of a focal lesion in the basal ganglia of children with above features should prompt the diagnosis of ectopic germinoma, hence obviating delay in the diagnosis of a malignancy with an otherwise favourable prognosis (Ozalame et al., 2006).

Some basal ganglia germinomas are difficult to diagnose in early stage of disease due to slow clinical course, vague initial presentation, indiscernible mass lesion on imaging and absence of early raised intracranial pressure (Machara et al., 1983; Tamaki et al., 1990; Kim, 2002). Cerebral hemiatrophy can precede the imaging depiction of the off-midline mass (Sartori et al., 2007).

CT finding of germinoma is characterized by an irregularly defined, slightly high-density area or isodensity frequently accompanied by central low-density areas without significant mass effect. The tumors showed mild to moderate and non-homogeneous enhancement (Tamaki et al., 1990; Inoue et al., 1979). MRI images shows tumor exact location, associated ipsilateral cerebral hemisphere and brain stem atrophy (Kim, 2002). Immunohistochemically, germs cell tumour of the basal ganglia has a high propensity of containing other components like choriocarcinoma, endodermal sinus tumor, and embryonal carcinoma (Zhang et al., 2007; Tamaki et al., 1990). These will cause variable MRI signal changes.

Early diagnosis of germinoma and subsequent radiotherapy may prevent unnecessary surgery as they are radiosensitive even at small doses (Machara et al., 1983; Tamaki et al., 1990; Inoue et al., 1979).

(16) Silver Russell Syndrome

This is growth failure originating from spontaneous growth hormone abnormality evident from birth. Incidence is 1 per 3000-100,000 population with equal sex ratio. Silver-Russell syndrome (SRS) originally was described by Silver and colleagues in 1953 and soon afterwards, by Russell in 1954 (Ferry, 2011).

SRS is chromosomal in some patients with maternal uniparental disomy of chromosome 7, and the possibility of imprinting (i.e inheriting 2 copies of maternal chromosome 7, with no paternal contribution) (Ferry, 2011).

Clinically patient has triangular face with down-turned corners of the mouth, frontal bossing (phenotypic facial dysmorphism), asymmetrical growth and skeletal maturation. Others are intrauterine growth retardation, difficulty in feeding, failure to thrive, postnatal growth retardation, normal intelligence or learning disability. Facial dysmorphism is observed, with normal head circumference but because length usually is less than normal, the head appears disproportionately large. Assymetrical skeletal maturation can lead to dwarfism or hemihypertrophy. Camptodactyly (fixed flexion of digits) or clinodactyly (incurving) of one or more fingers may be seen (Ferry, 2011).

Growth hormone therapy is used in a SRS child without adequate catch-up growth by age two (Ferry, 2011).

(17) Haberland Syndrome (Fishman Syndrome)

Haberland syndrome (HS) otherwise called Encephalocraniocutaneous lipomatosis (ECCL) is a rare, congenital, neurocutaneous disorder with ipsilateral ophthalmologic/neurologic malformations. It is a unilateral lipomatous cutaneous neoplasms devoid of hair (Sergiusz, 2012; Rubegni et al., 2003). Haberland and Perou first described the disorder in 1970 in the clinical and necropsy findings of a 51-year-old man who has epilepsy and mental retardation (Sergiusz, 2012).

The pathogenesis of Haberland syndrome remains unknown but dysgenesia of the cephalic neural crest and the anterior neural tube is a most widely accepted theory (Sergiusz, 2012). Clinical features are unilateral lipomatous hamartomata of the scalp, eyelid, and outer globe of the eye, ipsilateral porencephalic cysts with cortical atrophy, cranial asymmetry, marked developmental delay and mental retardation. Differential diagnosis are other mosaic neurocutaneous phenotypes such as Delleman syndrome, Schimmelpenning syndrome, Goltz syndrome, Goldenhar syndrome and Proteus syndrome (Rubegni et al., 2003).

(18) Miscellaneous Causes

A vascular cause of cerebral hemiatrophy (hypoplasia) was first proposed in 1860 (Sharma et al., 2006). A vascular anomaly occurring in very early gestation (five or six weeks) may result in a major defect in brain development whereas later occurrence may produce more localized lesions (Sharma et al., 2006).

Chridtopher-Rodgman et al. (2011) published a case presentation of an 18-year-old female emigrant from Ghana who presented with complaints of seizures with aetiogenesis linked to cerebral malaria suffered at 13year of life. They hypothesized that the cerebral malaria with related vascular occlusion were reasons for the acquired cerebral changes with homolateral hypertrophy of the skull and sinuses (Ozalame et al., 2006).

## 5. Conclusion

Childhood cerebral hemiatrophy is an uncommonly encountered clinical entity. Our two newly discovered cases would have escaped recognition but for exploits of neuro-imagings especially MRI. The authors intend to emphasize the revelance of increasing availability of neuro-imaging modalities in sub-saharan Africa as their findings may immensely dilute entities hitherto regarded as uncommon. Literature review gave an extensive gamut of differential diagnoses of childhood cerebral hemiatrophy including neoplasm that ordinarily is known to provoke peri-tumoral edema
